# A Microfluidic-Based Fabry Pérot Gas Sensor

**DOI:** 10.3390/mi7030036

**Published:** 2016-02-25

**Authors:** Jin Tao, Qiankun Zhang, Yunfeng Xiao, Xiaoying Li, Pei Yao, Wei Pang, Hao Zhang, Xuexin Duan, Daihua Zhang, Jing Liu

**Affiliations:** 1 State Key Laboratory of Precision Measurement Technology and Instruments, School of Precision Instruments and Opto-Electronics Engineering, Tianjin University, Tianjin 300072, China; taojin@tju.edu.cn (J.T.); zqkty@tju.edu.cn (Q.Z.); weipang@tju.edu.cn (W.P.); haozhang@tju.edu.cn (H.Z.); xduan@tju.edu.cn (X.D.); dhzhang@tju.edu.cn (D.Z.); 2 State Key Laboratory for Mesoscopic Physics, School of Physics, Peking University, Beijing 100044, China; yfxiao@pku.edu.cn; 3 Key Laboratory of Optoelectronics Information Technology, School of Precision Instrument and Opto-Electronics Engineering, Tianjin University, Tianjin 300072, China; xiaoyingli@tju.edu.cn; 4 School of Materials Science and Engineering, Tianjin University, Tianjin 300072, China; pyao@tju.edu.cn

**Keywords:** micro gas sensor, micro Fabry-Pérot cavity, optical fiber, microfluidic channel, MEMS

## Abstract

We developed a micro-gas detector based on a Fabry-Pérot (FP) cavity embedded in a microfluidic channel. The detector was fabricated in two steps: a silicon substrate was bonded to a glass slide curved with a micro-groove, forming a microfluidic FP cavity; then an optical fiber was inserted through a hole drilled at the center of the groove into the microfluidic FP cavity, forming an FP cavity. The light is partially reflected at the optical fiber endface and the silicon surface, respectively, generating an interference spectrum. The detection is implemented by monitoring the interference spectrum shift caused by the refractive index change of the FP cavity when a gas analyte passes through. This detection mechanism (1) enables detecting a wide range of analytes, including both organic and inorganic (inertia) gases, significantly enhancing its versatility; (2) does not disturb any gas flow so that it can collaborate with other detectors to improve sensing performances; and (3) ensures a fast sensing response for potential applications in gas chromatography systems. In the experiments, we used various gases to demonstrate the sensing capability of the detector and observed drastically different sensor responses. The estimated sensitivity of the detector is 812.5 nm/refractive index unit (RIU) with a detection limit of 1.2 × 10^−6^ RIU assuming a 1 pm minimum resolvable wavelength shift.

## 1. Introduction

Gas detectors attract a lot of research interest due to their wide applications in the areas of environmental monitoring [1,2], homeland security [3], anti-terrorism [4], industrial quality control [5,6,7], *etc.* So far, various stand-alone gas detectors, such as surface acoustic wave detectors [8], chemiresistor detectors [9,10,11,12,13,14,15], grating-based optical gas detectors [16], surface Plasmon resonance gas detectors [17,18] and opto-thermal gas detectors [19], have been successfully developed and made commercially available. However, most of them either respond to very few analytes, significantly limiting their application fields [20,21], or respond similarly to a great amount of analytes, so that one cannot be differentiated from others, which can hardly satisfy the increasing demands of applications requiring the identification of a wide range of analytes in a complex mixture.

One of the most promising solutions to this problem is to combine gas detector(s) with a gas chromatography system (GC), to separate gas mixtures by their different velocities when traveling through the separation column [22]. Thus, a detector can be installed at the elution end of the separation column to detect individual analytes sequentially as they elute out in succession. The detector that can be used in a GC usually possesses several features: (1) it can detect a reasonable amount of analytes; (2) it has a flow-through structure and can be easily connected to the GC; and (3) it has a fast sensing response so that it can complete the detection of one analyte before another one elutes out (it takes several seconds to minutes for analytes to elute out from the GC system). Most of the stand-alone detectors are not readily applicable in GC systems because they do not have one or more of the aforementioned feature(s). 

The traditional GC detectors are generally divided into destructive and non-destructive detectors. The destructive detectors, such as mass spectrometry and the flame ionization detector, usually detect the current change caused by the ionization of analytes [23,24], and thus the analytes are destroyed after detection. This feature is not desirable for the applications that require multiple detectors to work in concert to acquire complimentary information of the analyte for improving system performance, such as to promote the analyte identification rate in a multi-dimensional GC [22,25,26] and to automate system control in smart GCs [27,28]. In contrast, non-destructive detectors do not destroy analytes during the detection and therefore are much more flexible and applicable than destructive detectors in the system design and integration. One of the most widely used non-destructive detectors in GC is the thermal conductivity detector (TCD), which identifies the analyte by comparing the heat loss caused by passing through the analyte with the loss caused by passing through the carrier gas per unit time [29]. Consequently, it not only keeps the analyte intact during the detection, but also is a universal detector which responds to most of the analytes except the ones that have the same heat capacity as the carrier gas [30]. This capability is very useful for quick sample identification and pre-detection in a multi-detector GC system. Nevertheless, traditional TCD usually has large dead volume, leading to broadened analyte peaks and large sample consumption, and has to work in an elevated temperature, resulting in high power consumption and potential hazards for detecting flammable and explosive gases, which is not desirable for being integrated in miniaturized GC systems designed to satisfy the dramatically increased quick on-site detection demands in recent years [31].

Recently, a lot of microstructured gas detectors have been developed to be integrated with micro-GC and/or microfluidic systems for on-site quick gas detection [32,33,34,35]. One of the promising detection schemes is based on the Fabry-Pérot (FP) cavity, which is also widely used in the fields of biosensing, temperature, strain, and humidity sensing because of its simple fabrication and measurement setup. There are mainly two ways to form the FP cavity: using a polymer layer or using the microfluidic channel itself. The polymer layer, either deposited at the end of an optical fiber [33,36] or the bottom of the microfluidic channel [34,37], interacts with the gas analyte and results in the change of its refractive index (RI) or/and thickness, which causes the shift of the interference spectrum. The usage of a polymer improves the sensitivity and selectivity of the sensors; however, it limits the detectable gases to being the ones that have interactions with the polymer, greatly restricting its applications. One the other hand, the microfluidic channel can also form an FP cavity with its inner surfaces as the reflective surfaces. When the analyte is traveling through the microfluidic channel, the RI of the FP cavity changes, leading to the shift of the interference spectrum. This configuration has been used in biosensing [38,39,40], pressure sensing [41,42], and temperature sensing [43], but it has not yet, to the best of our knowledge, been used in universal gas sensing.

**Figure 1:**
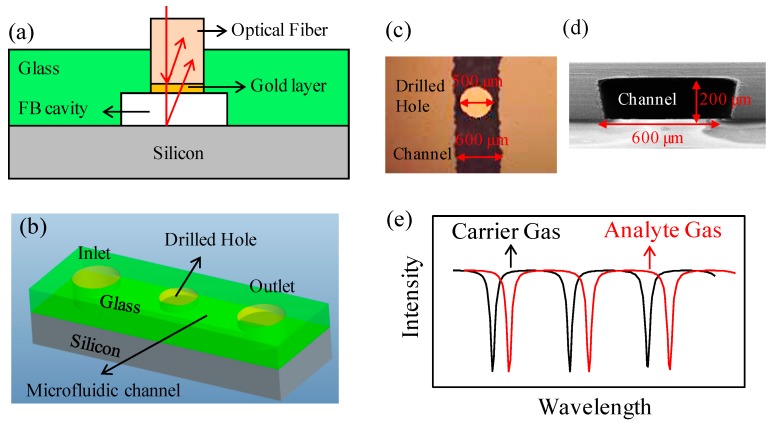
(**a**) Schematic diagram of the cross-section of the microfluidic-based FP-gas detector. Red arrows represent the light beam which was coupled into the optical fiber and reflected at the gold layer and silicon surface, respectively. (**b**) Three-dimensional diagram of the microfluidic-based FP gas detector. (**c**) Optical microscopy image of the top view of the microfluidic channel. (**d**) SEM image of the sectional view of the microfluidic channel. (**e**) Exemplary interference spectrum shift caused by the analyte gas.

In this paper, we demonstrated the possibility of the microfluidic-based FP sensor for a non-destructive and universal gas detector which has the potential to be further integrated with miniaturized/portable GC systems. The detector, as shown in Figure 1a, is fabricated by inserting a single-mode optical fiber into a hole drilled at the center of a micro-groove curved on a glass slide which is then bonded to a silicon substrate, forming a microfluidic channel. The light coupled into the optical fiber partially reflects at the optical fiber endface and the silicon surface, respectively, generating an interference spectrum.

When an analyte travels through, the RI of the FP cavity changes. The shift of the resonant wavelength (interference spectrum) is linearly related to the change of the RI of the FP cavity and hence the passage of the analyte. Therefore, by monitoring the interference spectrum shift, the kinetic information of the analyte can be obtained.

There are several unique advantages of the proposed detector. First of all, the detector detects the RI change at the detection point in the microfluidic channel, and, therefore, it can detect an analyte that presents an RI difference from the carrier gas larger than its detection limit, validating its capability of detecting most analytes including organic, inorganic and even inertia gases. Second, since it detects the RI change of the FP cavity, whose two reflectance surfaces are both flush with the inner surface of the microfluidic channel, the detector does not destroy or induce any disturbance to the gas flow, which has the potential to be used in combination with other detectors in a series to improve the detection performance. Third, the detector is embedded in a microfluidic channel, forming a flow-through structure to minimize its dead volume, and at the same time does not involve any analyte absorption/desorption processes, significantly reducing the peak-broadening and speeding up the detection response compared to TCD and other detectors. This merit is extremely important for miniaturized GC to increase its detection efficiency (the number of analytes detected per unit time), because sharp peaks enable it to resolve more analytes per unit time than conventional GC and hence it may resolve as many analytes as a conventional one does in a much shorter time. Fourth, the flow-through structure of the detector allows it to be readily connected to the fluidic channel of the GC. Fifth, it does not need any heating elements or additional gas supply for the proposed detector to be fully functional, which favors the whole system’s miniaturization and portability. Last, the fabrication process is compatible with the micro-fabrication process, further lowering the manufacturing cost and benefiting the mass production.

We carried out the initial calibration and characterizations of the proposed gas detector to demonstrate its detection capability. In our experiments, we coated gold layers of various thicknesses at the endfaces of different optical fibers to increase the reflectivity at the optical fiber-air interface, so that an optimal interference spectrum can be obtained. Then, the performance of the detector was tested by various analytes with different physical and chemical properties, which, as expected, responded linearly to the amount of the RI change caused by its exposure to the analyte with a sensitivity of 812 nm/RIU and an estimated detection limit of 1.2 × 10^−6^ RIU assuming that the minimum resolvable wavelength shift was 1 pm, corresponding to the detection limit of 2800 ppm for C_2_H_4_.

## 2. Experimental

### 2.1. Detector Fabrication

The two- and three-dimensional schematic structures of the detector are shown in Figure 1a,b, respectively, the two reflectance surfaces of which were the endface of an optical fiber and the surface of the silicon substrate, respectively. The optical fiber was inserted into a hole (see Figure 1c) drilled at the center of a micro-groove etched on a glass slide which was then anodic-bonded with a silicon substrate, forming a sealed micro-fluidic channel (its cross-sectional dimension was 600 μm wide by 200 μm deep, as presented in Figure 1d). The optical fiber endface was aligned with the inner surface of the channel to avoid any potential disturbance to the fluidic flow. The hole was then sealed by silicone rubber to prevent any gas leakage. The endface of the optical fiber was coated with gold by the physical vapor deposition method to increase the reflectivity. When an analyte gas was pumped through, the RI of the FP cavity changed, resulting in the interference spectrum shift as shown in Figure 1e.

### 2.2. Experimental Setup

**Figure 2:**
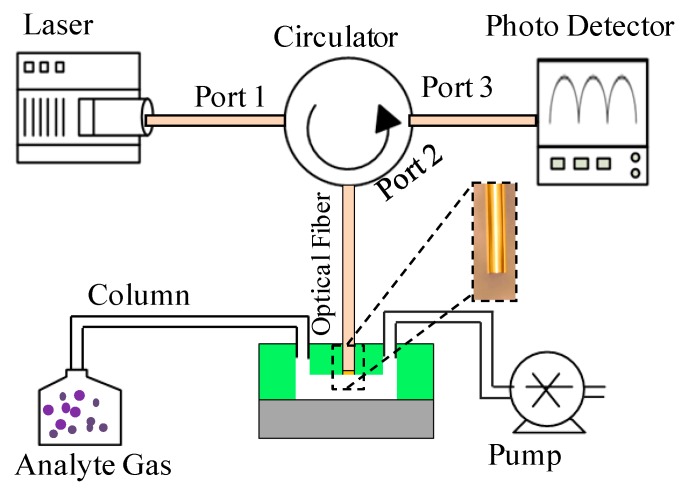
Schematic of the test system. The inset shows the image of the optical fiber.

The whole test system was composed of the optical measurement part and gas delivery part, as shown in Figure 2. The optical measurement part consisted of a laser source, an optical circulator and a photon detector. The laser was scanned from 1510 to 1590 nm at a frequency of 2 Hz with a spectral resolution of 1 pm. The laser output was coupled into Port 1 of the circulator and delivered into the sensing optical fiber through Port 2. The reflected light from the sensing probe was coupled back to Port 2 and delivered into Port 3 which was connected to a photo detector. The light intensity was monitored by a homemade LabVIEW program at a recording rate of 20 kHz. The gas analytes were prepared individually by drawing the pure analyte into a gas-tight syringe until it reached the pressure equilibrium. The outlet of the syringe was connected to the inlet of the microfluidic channel through a capillary column. A syringe pump was used to pump the gas analyte from the syringe into the microfluidic channel at a flow rate of 10 μL/min.

## 3. Results and Discussion

The microfluidic-based FP gas detector monitors the interference spectrum shift of the microfluidic FP cavity caused by its RI change when the gas analyte passes through. This section first investigated the effect of the gold coating on the quality of the interference spectrum. Then, various gas analytes, which include organic gases such as CH_4_, C_2_H_4_ and C_3_H_6_O, inorganic gases such as NH_3_, N_2_O, and CO_2_, and the inertia gas of He, were used to characterize the detector performance and demonstrate its versatility. Such a wide range of detectable gases can be hardly covered by a single detector.

### 3.1. Gold Coating Calibration

The reflectivity of the two surfaces of the FP cavity, the optical fiber endface and the silicon surface, decide the quality of the interference spectrum and thus the detection limit of the proposed detector. Here, we used gold coating at the optical fiber endface as a model method to increase its reflectivity while keeping the silicon surface uncoated due to the necessity to simplify the fabrication process of the microfluidic channel. The theoretical calculation was carried out to analyze the effect of the reflectivity of the optical fiber endface on the interference spectrum when the reflectivity of the silicon surface was kept at a constant value of 0.3.

**Figure 3:**
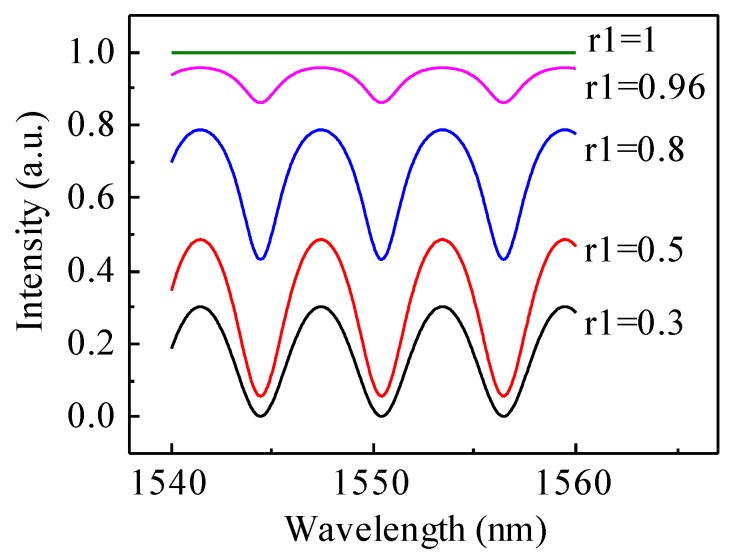
Theoretical interference spectra of the FP cavity when *r*_2_ is set to be 0.3.

The normalized reflectance intensity *R* of the FP detector can be described by the following equation:

(1) $$R=\frac{I_{r}}{I_{e}}=1-\frac{t_{1}^{2}t_{2}^{2}}{{\left(1-r_{1}r_{2}\right)}^{2}+4r_{1}r_{2}\sin^{2}(kd)}$$

where *I_r_* and *I_e_* are the reflectance and input light intensity, respectively; t_1_*/t*_2_ and *r*_1_*/r*_2_ are the transitivity and reflectivity of the optical fiber endface and silicon surface, respectively; *d* is the FP cavity length; and *k* is the wave vector. Based on Equation (1), the normalized reflected interference spectra are plotted in Figure 3 to visualize the effects of *r*_1_ on the sharpness and contrast of the interference (the value of *r*_2_ is set to be 0.3). The interference spectra correspond to five values of *r*_1_ of 0.3, 0.5, 0.8, 0.96 and 1. As expected, the sharpness of the resonant peaks increases when *r*_1_ increases, while the contrast reaches the maximum value when *r*_1_ equals 0.5.

**Figure 4:**
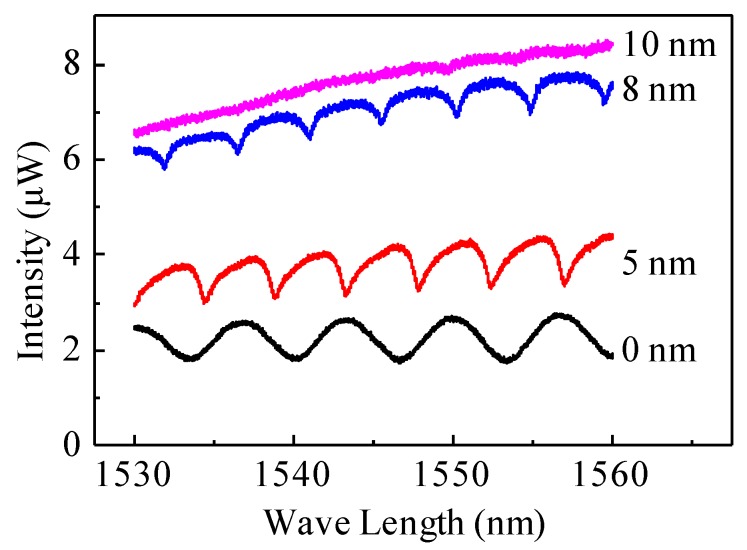
Experimental interference spectra of the FP cavities with various thicknesses of gold coating on the optical fiber enfaces.

We then carried out experimental tests in which gold layers with thicknesses of 5, 8 and 10 nm were coated on the endfaces of three optical fibers, respectively, whose resultant spectra are presented in Figure 4. The trend was generally consistent with the theoretical observation: when the thickness of the gold layer increased, leading to the increase of the reflectivity, the sharpness of the resonant peaks also increased. Nevertheless, the contrast of the spectrum generated by the optical fiber without any gold coating was similar to the contrast generated by the optical fiber with 5 nm gold coating, which may suggest that a larger contrast can be obtained by an optical fiber with a thinner gold layer. When the gold layer reached 10 nm, the light was totally reflected at the optical fiber endface. Since the 5 nm gold coating was the thinnest coating that could be accurately deposited in our lab, and it generated an interference spectrum with reasonably sharp resonant peaks and large contrast at the same time, the optical fiber endface was deposited with 5-nm-thick gold coating in the following experiments. In future development, other methods will be used to increase the reflectivity of both reflectance surfaces to greatly improve the quality of the FP cavity and also that of the detection limit. 

### 3.2. Real-Time Response

**Figure 5:**
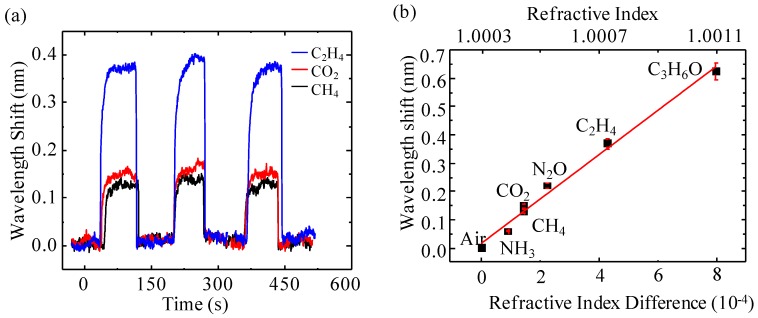
(**a**) Real-time responses of the sensor to C_2_H_4_, CO_2_ and CH_4_. (**b**) The wavelength shift corresponding to the RI change caused by exposure to various analytes.

We calibrated the responses of the detector to various pure gas analytes which are presented in Figure 5. At the beginning, the ambient air was pumped in the microfluidic channel as the carrier gas (RI = 1.000292) to establish the baseline of the sensor, after which pure gas analyte was injected in. Since the RI of the gas analyte was different from the carrier gas, the RI of the FP cavity changed when the gas analyte passed through, resulting in the shift of the interference spectrum. When the interference shift reached the maximum equilibrium, the carrier gas was switched back into the microfluidic channel to purge out the gas analyte. Consequently, the signal of the detector returned back to the baseline. Each gas analyte was tested by the aforementioned procedures multiple times to demonstrate the detector’s repeatability and reliability. Figure 5a shows the real-time responses of our detector to three types of gas analytes: C_2_H_4_, CO_2_ and CH_4_, whose RIs are 1.0007198, 1.000449 and 1.000444, respectively. Since it generated the biggest RI change of 4.28 × 10^−4^ compared to the other two analytes, C_2_H_4_ had the largest interference shift of around 0.38 nm. On the other hand, CO_2_ and CH_4_ caused a RI change of 1.57 × 10^−4^ and 1.52 × 10^−4^, respectively, and thus the detector had an interference shift of 0.15 nm and 0.14 nm, respectively. The detector had sharp “on” and “off” response signals to all the analytes (around 0.5 to 1 s, limited by the sampling rate), which is because it has minimum dead volume and does not involve any analyte absorption/desorption processes. The quick response is very important for the micro-GC, because sharp peaks enable it to resolve more analyte peaks per unit time than broad peaks do, thus improving its analysis efficiency. Additionally, the wavelength shifts of the detector upon its exposure to three more analytes of NH_3_, N_2_O, and C_3_H_6_O were also recorded and are presented in Figure 5b, which shows the relationship between the interference shifts of the detector with both the absolute RI of the gas analyte and the RI difference of the gas analyte from the carrier gas. As expected, the wavelength shift is linearly proportional to both the absolute RI and the RI difference from air of the analyte gas, from which the sensitivity of the detector is estimated to be 812.5 nm/RIU. Since the wavelength stability of the laser source is around 1 pm, the detection limit of the detector is estimated to be a change of 1.2 × 10^−6^ RIU, which is similar to the sensors reported in [17,44,45]. Although other undesirable noises, such as the syringe pump, caused vibration, the static noise (2 pm) and the temperature fluctuation degraded it to around 10 pm, and the noise level can be minimized to 1 pm by controlling the system parameters well.

**Figure 6:**
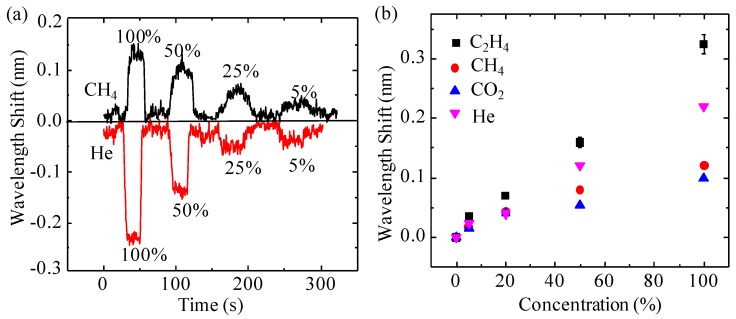
(**a**) Real-time responses of the sensor to CH_4_ and He with concentrations of 100%, 50%, 25% and 5%. (**b**) Concentration-dependent wavelength shift upon exposure to C_2_H_4_, CH_4_, CO_2_ and He with concentrations of 100%, 50%, 25% and 5%.

The detector was also calibrated by analytes with concentrations ranging from 100% to 5% by mixing a single analyte with the carrier gas. Figure 6a is the real-time response of the detector to CH_4_ and helium (He) with the concentrations of 100%, 50%, 25% and 5%. The absolute values of the interference spectrum shifts of the detector to both analytes declined gradually as the concentrations of both gas analytes dropped from 100% to 5%, and the RI values approached that of the carrier gas. Additionally, the interference spectrum of the detector shifted to a longer wavelength when it was exposed to CH_4_, while it shifted to a shorter wavelength when it was exposed to He. The phenomenon can be explained by Equation (1): when the RI change, Δ*n*, is positive/negative, the interference spectrum shift is positive/negative (shift to longer/shorter wavelength). Consequently, CH_4_/He, whose RI is larger/smaller than the RI of the carrier gas (air), caused the interference shift to a longer/smaller wavelength. This feature can be used to compare the RI value of the analyte with the RI value of the carrier gas, which may be an important parameter for identifying an unknown gas. In Figure 6b, the values of the absolute interference spectrum shift at equilibrium for different concentrations of CO_2_, CH_4_, C_2_H_4_ and He are depicted, in which the absolute value of the wavelength shift increases linearly as the concentration of the analyte increases. From this figure, the sensitivity (detection limit) of the detector in terms of concentration is estimated to be 3.5 × 10^−4^ pm/ppm (2800 ppm) and 10^−4^ pm/ppm (10,000 ppm) for C_2_H_4_ and CO_2_, respectively, which has the maximum (C_2_H_4_) and minimum (CO_2_) RI difference from the carrier gas, respectively. 

## 4. Materials and Methods

All of the analyte gases used in the experiment were purchased from Best Gas (Tianjin, China) and had purity greater than 99.9%. Pyrex 7740 glass wafer was used for anode bonding with the silicon wafer by the wafer bonder (SB6/8, SUSS MicroTec, Garching, Germany). A fiber sensor system (SM125, Micron Optics Inc., Hackettstown, NJ, USA) was used as the laser source. Single-mode fibers (SMF-28) were purchased from Corning (New York, NY, USA). Universal quick seal column connectors (Part No. 23627) were purchased from Sigma (St. Louis, MO, USA). The syringe pump (Model NO: NE1000) was purchased from New Era Pump System (Farmingdale, NY, USA) The capillary column (Model NO: 160-2255 DEAC1) was purchased from Agilent Technologies (Santa Rosa, CA, USA). The silicone rubber used to seal the column and the FP cavity was purchased from NanDa Inc., Tianjin, China.

## 5. Conclusions

We developed a non-destructive and universal microfluidic-based FP gas detector. The detector was tested by various analytes with diversified chemical and physical properties with various concentrations, and the results show that it had a sensitivity of 812.5 nm/RIU and a detection limit of 1.2 × 10^−6^ RIU, assuming the minimum resolvable wavelength shift was 1 pm which is comparable to other optical sensors [36,38,40,41,42]. The detector senses the RI change in the microfluidic channel where the detector is embedded when the gas analyte passes through. Therefore, it can detect any type of analyte that has a different RI from that of the carrier gas, significantly improving its versatility, which is highly valuated by the quick on-site sample identification applications. The non-destructive detection mechanism and flow-through structure allow the detector to be installed upstream of other detectors to provide complementary information. In addition, the detector also has a fast response due to its minimized dead volume and the disuse of any gas-absorptive materials, which suggests its potential for being used in GC systems. The future work will be focused on promoting the performance of the detector by increasing the quality of the micro-FP cavity to improve the interference spectrum resolution and thus the detection limit.

## References

[1] Fine G.F., Cavanagh L.M., Afonja A., Binions R. (2010). Metal oxide semi-conductor gas sensors in environmental monitoring. Sensors.

[2] Martinelli G., Carotta M.C., Ferroni M., Sadaoka Y., Traversa E. (1999). Screen-printed perovskite-type thick films as gas sensors for environmental monitoring. Sens. Actuators B Chem..

[3] Moore D.S. (2007). Recent advances in trace explosives detection instrumentation. Sens. Imaging.

[4] Wang J. (2007). Electrochemical Sensing of Explosives. Electroanalysis.

[5] Bourrounet B., Talou T., Gaset A. (1995). Application of a multi-gas-sensor device in the meat industry for boar-taint detection. Sens. Actuators B Chem..

[6] Schweizer-Berberich P.M., Vaihinger S., Göpel W. (1994). Characterisation of food freshness with sensor arrays. Sens. Actuators B Chem..

[7] Ólafsdóttir G., Martinsdóttir E., Oehlenschläger J., Dalgaard P., Jensen B., Undeland I., Mackie I.M., Henehan G., Nielsen J., Nilsen H. (1997). Methods to evaluate fish freshness in research and industry. Trends Food Sci. Technol..

[8] Ricco A.J., Martin S.J., Zipperian T.E. (1985). Surface acoustic wave gas sensor based on film conductivity changes. Sens. Actuators B Chem..

[9] Abraham J.K., Philip B., Witchurch A., Varadan V.K., Reddy C.C. (2004). A compact wireless gas sensor using a carbon nanotube/PMMA thin film chemiresistor. Smart Mater. Struct..

[10] Paul R.K., Badhulika S., Saucedo N.M., Mulchandani A. (2012). Graphene nanomesh as highly sensitive chemiresistor gas sensor. Anal. Chem..

[11] Ho K.C., Tsou Y.H. (2001). Chemiresistor-type NO gas sensor based on nickel phthalocyanine thin films. Sens. Actuators B Chem..

[12] Yamazoe N., Sakai G., Shimanoe K. (2003). Oxide semiconductor gas sensors. Catal. Surv. Asia.

[13] Yamazoe N. (1991). New approaches for improving semiconductor gas sensors. Sens. Actuators B Chem..

[14] Comini E., Faglia G., Sberveglieri G., Pan Z., Wang Z.L. (2002). Stable and highly sensitive gas sensors based on semiconducting oxide nanobelts. Appl. Phys. Lett..

[15] Simon I., Bârsan N., Bauer M., Weimar U. (2001). Micromachined metal oxide gas sensors: Opportunities to improve sensor performance. Sens. Actuators B Chem..

[16] Zhou B., Chen Z., Zhang Y., Gao S., He S. (2014). Active Fiber Gas Sensor for Methane Detecting Based on a Laser Heated Fiber Bragg Grating. IEEE Photonics Technol. Lett..

[17] Bingham J.M., Anker J.N., Kreno L.E., Van Duyne R.P. (2010). Gas Sensing with High-Resolution Localized Surface Plasmon Resonance Spectroscopy. JACS.

[18] Sharma A.K., Jha R., Gupta B.D. (2007). Fiber-optic sensors based on surface plasmon resonance: A comprehensive review. IEEE Sens. J..

[19] Rosengren L.G. (1973). An opto-thermal gas concentration detector. Infrared Phys..

[20] Morrison S.R. (1987). Selectivity in semiconductor gas sensors. Sens. Actuators.

[21] Coles G.S.V., Williams G., Smith B. (1991). Selectivity studies on tin oxide-based semiconductor gas sensors. Sens. Actuators B Chem..

[22] Liu J., Sun Y., Howard D.J., Frye-Mason G., Thompson A.K., Ja S.J., Wang S.K., Bai M., Taub H., Almasri M., Fan X. (2010). Fabry-Pérot cavity sensors for multipoint on-column micro gas chromatography detection. Anal. Chem..

[23] Hobbs P.J., Misselbrook T.H., Pain B.F. (1995). Assessment of Odours from Livestock Wastes by a Photoionization Detector, an Electronic Nose, Olfactometry and Gas Chromatography-Mass Spectrometry. J. Agric. Eng. Res..

[24] McWilliam I.G., Dewar R.A. (1958). Flame ionization detector for gas chromatography. Nature.

[25] Marriott P.J., Chin S.T., Maikhunthod B., Schmarr H.G., Bieri S. (2012). Multidimensional gas chromatography. Trends Anal. Chem..

[26] Seeley J.V. (2012). Recent advances in flow-controlled multidimensional gas chromatography. J. Chromatogr. A.

[27] Liu J., Seo J.H., Li Y., Chen D., Kurabayashi K., Fan X. (2013). Smart multi-channel two-dimensional micro-gas chromatography for rapid workplace hazardous volatile organic compounds measurement. Lab Chip.

[28] Chen D., Seo J.H., Liu J., Kurabayashi K., Fan X. (2013). Smart Three-Dimensional Gas Chromatography. Anal. Chem..

[29] Cruz D., Chang J.P., Showalter S.K., Gelbard F., Manginell R.P., Blain M.G. (2007). Microfabricated thermal conductivity detector for the micro-ChemLab™. Sens. Actuators B Chem..

[30] Simon I., Arndt M. (2002). Thermal and gas-sensing properties of a micromachined thermal conductivity sensor for the detection of hydrogen in automotive applications. Sens. Actuators A Phys..

[31] Kuo J.T., Yu L., Meng E. (2012). Micromachined thermal flow sensors—A review. Micromachines.

[32] Zhong Q., Steinecker W.H., Zellers E.T. (2009). Characterization of a high-performance portable GC with a chemiresistor array detector. Analyst.

[33] Liu J., Sun Y., Fan X. (2009). Highly versatile fiber-based optical Fabry-Pérot gas sensor. Opt. Express.

[34] Reddy K., Guo Y., Liu J., Lee W., Oo M.K.K., Fan X. (2011). On-chip Fabry-Pérot interferometric sensors for micro-gas chromatography detection. Sens. Actuators B Chem..

[35] Hossein-Babaei F., Paknahad M., Ghafarinia V. (2012). A miniature gas analyzer made by integrating a chemoresistor with a microchannel. Lab Chip.

[36] Gao R., Jiang Y., Ding W., Wang Z., Liu D. (2013). Filmed extrinsic Fabry-Perot interferometric sensors for the measurement of arbitrary refractive index of liquid. Sens. Actuators B Chem..

[37] Reddy K., Guo Y., Liu J., Lee W., Oo M.K.K., Fan X. (2012). Rapid, sensitive, and multiplexed on-chip optical sensors for micro-gas chromatography. Lab Chip.

[38] Wei T., Han Y., Li Y., Tsai H.L., Xiao H. (2008). Temperature-insensitive miniaturized fiber inline Fabry-Perot interferometer for highly sensitive refractive index measurement. Opt. Express.

[39] Lin C.H., Jiang L., Xiao H., Chai Y.H., Chen S.J., Tsai H.L. (2009). Fabry-Perot interferometer embedded in a glass chip fabricated by femtosecond laser. Opt. Lett..

[40] Tian Y., Wang W., Wu N., Zou X., Guthy C., Wang X. (2011). A miniature fiber optic refractive index sensor built in a MEMS-based microchannel. Sensors.

[41] Xiao G.Z., Adnet A., Zhang Z., Sun F.G., Grover C.P. (2005). Monitoring changes in the refractive index of gases by means of a fiber optic Fabry-Perot interferometer sensor. Sens. Actuators A Phys..

[42] Duan D.W., Rao Y.J., Zhu T. (2012). High sensitivity gas refractometer based on all-fiber open-cavity Fabry-Perot interferometer formed by large lateral offset splicing. JOSA B.

[43] Kou J.L., Feng J., Ye L., Xu F., Lu Y.Q. (2010). Miniaturized fiber taper reflective interferometer for high temperature measurement. Opt. Express.

[44] Maharana P.K., Jha R., Padhy P. (2015). On the electric field enhancement and performance of SPR gas sensor based on graphene for visible and near infrared. Sens. Actuators B Chem..

[45] Goyal A.K., Pal S. (2015). Design and simulation of high-sensitive gas sensor using a ring-shaped photonic crystal waveguide. Phys. Scr..

